# Temporal relationship between inflammation and metabolic disorders and its influence on atherosclerotic cardiovascular disease

**DOI:** 10.3389/fcvm.2025.1664865

**Published:** 2026-03-02

**Authors:** Zhihui Liu, Huayu Sun, Hongmin Liu, Jintao Tao, Yutong Wu, Yixiu Chen, Yicheng Liao, Mo Zhang, Yufeng Bian, Renjie Fu, Yajing Liang, Wenchao Yao, Shouling Wu, Yuntao Wu

**Affiliations:** 1 Department of Cardiology, Kailuan General Hospital, Tangshan, China; 2 School of Graduate Studies, North China University of Science and Technology, Tangshan, China; 3 School of Public Health, North China University of Science and Technology, Tangshan, China; 4 University of Toronto, Toronto ON, Canada; 5 Graduate School, Hebei North University, Zhangjiakou, China

**Keywords:** inflammation, C-reactive protein, metabolic disorders, temporal relationship, longitudinal study

## Abstract

**Background:**

Atherosclerotic cardiovascular disease (ASCVD) is one of the conditions driven by imbalances in inflammatory and metabolic regulation. Although the temporal and causal relationships between inflammation and metabolic disorders are complex, the sequence in which they occur remains unclear. This study aimed to explore the temporal relationship between inflammation [high-sensitivity C-reactive protein (hsCRP)) and metabolic disorders (Continuous Metabolic syndrome score (cMets)] and their cumulative joint effects on ASCVD.

**Methods:**

This prospective cohort study was conducted in two parts. First, a longitudinal analysis was performed on 62,296 participants over a 4.05-year follow-up period using a cross-lagged panel model to investigate the directional relationship between hsCRP and cMetS. Second, after excluding 3,619 participants with a history of ASCVD, the remaining individuals were categorized into four groups to examine the time-weighted cumulative joint exposure of hsCRP and cMetS on the risk of ASCVD and its subtypes using Cox proportional hazards models.

**Results:**

There was a bidirectional relationship between hsCRP and cMetS, The robustness of the pathway from baseline hsCRP to follow-up cMetS (*β*_2_ = 0. 05661, 95% CI: 0. 04827–0. 06496, *P* < 0.01) exceeded that of the reverse pathway from baseline cMetS to follow-up hsCRP (*β*_1_ = 0. 05361, 95% CI: 0. 04511–0. 06212, *P* < 0.01), and the difference in coefficients was statistically significant (*P* < 0.01). The results of Cox proportional hazards regression analysis found that after adjusting for a variety of factors such as age and sex, compared with G1, G2, G3, and G4 had a higher risk of ASCVD HR (95% CI) were 1.45 (1.30, 1.62), 1.80 (1.66, 1.95), and 2.17 (1.98, 2.37), respectively. A significant interaction was observed between the exposure groups, 10-year ASCVD risk stratification, and age (*P*_interaction_ < 0.01). Stratified by age, the relative risk of ASCVD was found to be much higher in all groups for those aged <60 years than for those aged ≥60 years. Participants categorized as low-risk had greater relative increases in ASCVD relative risk than those in the intermediate- or high-risk groups.

**Conclusion:**

There is a bidirectional relationship between inflammation and metabolic disorders, with inflammation appearing to precede metabolic disorders. Concurrent exposure to both inflammation and metabolic disorders significantly increases the risk of ASCVD, particularly among individuals under 60 years of age and those classified as having a low risk of developing ASCVD in the next 10 years.

## Introduction

According to the 2021 Global Burden of Disease report, cardiovascular disease (CVD) one of the leading contributors to global disease burden and is the primary cause of death worldwide (1). With rapid population aging and shifts in lifestyle patterns, developing countries are projected to face persistently high burdens of CVD, with incidence and mortality rates expected to continue rising through 2050 (2). These trends underscore the urgent need for effective prevention and intervention strategies to mitigate the substantial public health impact.

Atherosclerotic cardiovascular disease (ASCVD) accounts for the majority of the global CVD burden. Existing studies point to ASCVD as one of the diseases caused by an imbalance between inflammation and metabolic regulation, and anti-inflammation and lipid modulation are theoretically two major therapeutic targets for intervening in ASCVD (3). However, the temporal order of inflammation and metabolic disorders remains unclear. Their interaction is complex and may form a vicious cycle that accelerates the onset and progression of ASCVD (4). Identifying the initiating trigger is therefore essential to breaking this cycle and improving cardiovascular health outcomes.

High-sensitivity C-reactive protein (hsCRP) is one of the most widely used clinical markers of systemic inflammation and has been recognized as an independent predictor of ASCVD. The Continuous Metabolic syndrome score (cMetS) is a composite index that quantitatively assesses the degree of metabolic disorders (5). Prior studies have independently examined the relationships between hsCRP and cMetS with ASCVD. A meta-analysis found that each standard deviation increase in hsCRP was associated with a 63% increased risk of coronary heart disease and a 44% increased risk of stroke (6). Additionally, multiple cohort studies have reported a positive linear association between cMetS and ASCVD risk (7–11). However, most existing research has focused on single time-point exposures, with limited evidence on their cumulative and joint effects.

To address this gap, based on data from the Kailuan cohort, our study aims to examine the temporal relationship between inflammation and metabolic disorders and their time-weighted cumulative joint impact on ASCVD risk. Specifically, we investigate the directional relationship between hsCRP and cMetS over time and assess how long-term co-exposure to elevated levels of both markers contributes to ASCVD development. These findings may provide important insights for improving ASCVD risk management and guiding preventive strategies.

## Methods

### Study population

**Figure 1:**
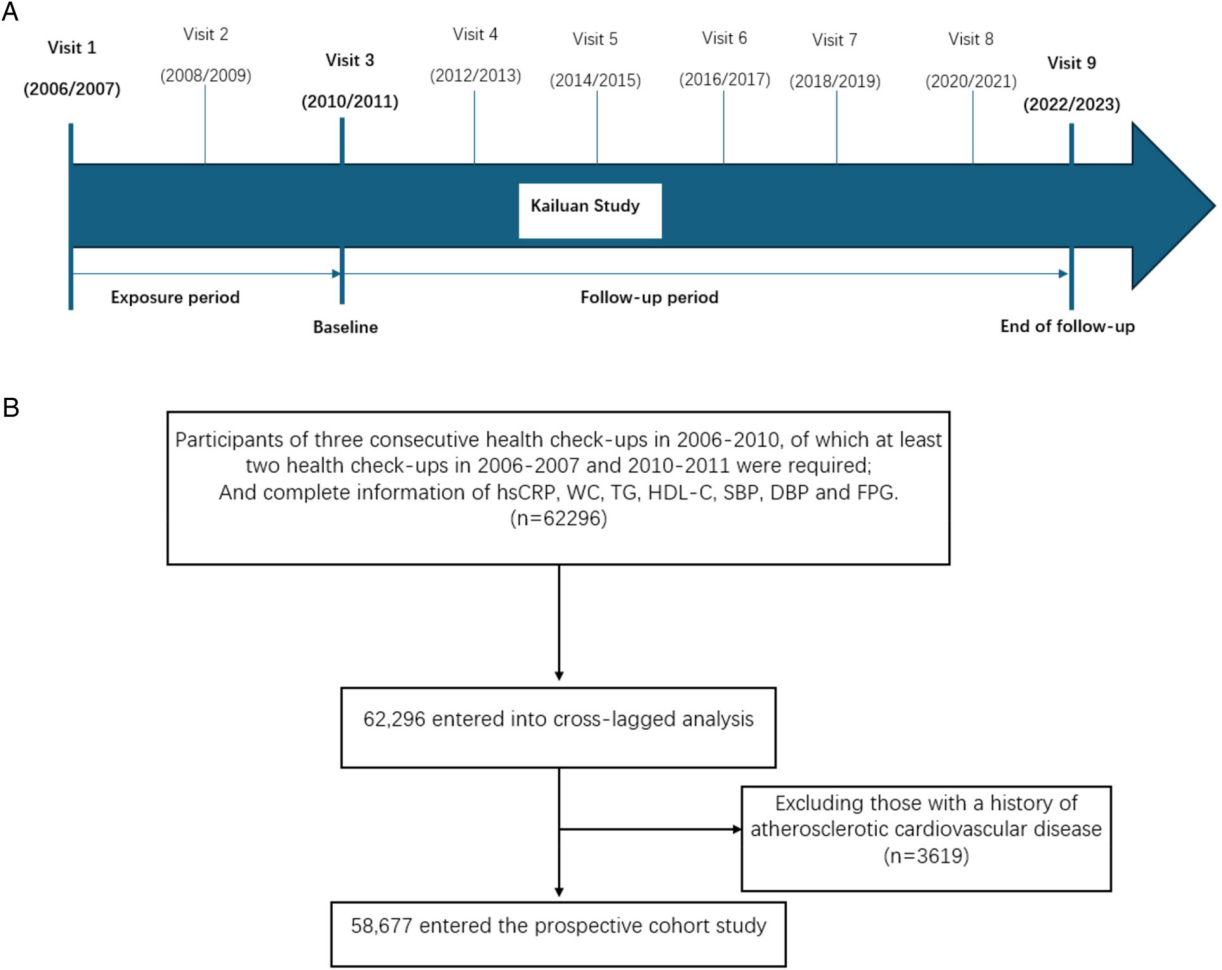
Study design and participant flow chart. **(A)** Design and strategy of the current study. **(B)** Flow chart of the study participants.

The Kailuan study (registration number ChiCTR-TNRC-11, 001, 489) is a large-scale prospective cohort study that was initiated in 2006 and is still ongoing. It focuses on investigating the risk factors for CVD and related diseases in a community-based cohort and the implementation of appropriate interventions. Starting from 2006, the Kailuan General Hospital and its 10 affiliated hospitals have conducted health examinations of both current and retired employees of the Kailuan Group every 2 years. In addition to routine follow-up assessments, the occurrence of adverse events, such as CVD, have been monitored annually. This study adhered to the principles of the Declaration of Helsinki and was approved by the Ethics Committee of Kailuan General Hospital. All participants provided written informed consent. This study selected from who participated in the Kailuan health check-ups in 2006–2010, at least two health check-ups in 2006–2007 and 2010–2011 were required and who had complete data of hsCRP, waist circumference (WC), triglycerides (TG), high-density lipoprotein cholesterol (HDL-C), systolic blood pressure (SBP), diastolic blood pressure (DBP), and fasting blood glucose (FPG) were used as observation subjects, with a total of 62,296 cases. Patients with a pre-existing history of ASCVD were excluded in 3619 cases, and 58,677 cases were included in the analysis. The specific study design for the current analysis is shown in Figure 1A, and the participant flow chart is shown in Figure 1B.

### Exposure assessment

1. *The Continuous metabolic syndrome score formula:*
cMetS=−3.1436+0.0258×WC+0.361×TG−0.9348×HDL−C+0.1224×MAP+0.1224×FPG
2. Time-weighted Average Cumulative exposure formula:
  $$\begin{array}{rl} & [(\mathrm{Inde}x_{2006/2007}+\mathrm{Inde}x_{2008/2009})/2\times \mathrm{Visi}t_{1-2}\\ & +(\mathrm{Inde}x_{2008/2009}+\mathrm{Inde}x_{2010/2011})/2\times \mathrm{Visi}t_{2-3}]/\mathrm{Visi}t_{1-3} \end{array}$$

Here, “Index2006/2007,” “Index2008/2009,” and “Index2010/2011” refer to the hsCRP or cMetS values measured during the health examinations in 2006–2007, 2008–2009, and 2010–2011, respectively. Meanwhile, “time1–2” and “time2-3” indicate the time intervals (in years) between two consecutive health examinations for each participant. Time-weighted Average Cumulative cMetS (CumcMetS) selects cut-off values based on RCS curves, and Time-weighted Average Cumulative hsCRP (CumhsCRP)selects cut-off values based on the corresponding clinical significance; the RCS curves for cMetS are displayed in Supplementary Figure 1:

G1: Average CumhsCRP below 3 mg/L and Average CumcMetS below 11.08;
G2: Average CumhsCRP equal to or above 3 mg/L and Average CumcMetS below 11.08;
G3: Average CumhsCRP below 3 mg/L and Average CumcMetS equal to or above 11.08;
G4: Average CumhsCRP equal to or above 3 mg/L and Average CumcMetS equal to or above 11.08;

### Biochemical measurements

Fasting (≥8 h) venous blood samples were collected between 7:00 and 9:00 a.m. on the day of the physical examination. Biochemical indicators were measured using a Hitachi 7600 automatic analyzer. Parameters includedFBG,TG, total cholesterol (TC), HDL-C, low-density lipoprotein cholesterol (LDL-C), and hsCRP. All measurements followed standardized procedures and were performed by certified laboratory technicians.

### Anthropometric and clinical measurements

Height, weight, WC, and blood pressure were measured. Body mass index (BMI) was calculated as weight (kg)/height (m^2^), and mean arterial pressure (MAP) was calculated as (SBP + 2  ×  DBP)/3. WC was measured at the midpoint between the lower margin of the rib cage and the top of the iliac crest, to the nearest 0.1 cm. Trained nurses measured height and weight between 7:30 and 9:00 a.m., with subjects barefoot, hatless, and wearing light clothing. Height and weight were measured to the nearest 0.1 cm and 0.1 kg, respectively. Blood pressure was measured on the brachial artery using a calibrated desktop mercury sphygmomanometer. SBP and DBP were defined by Korotkoff phase I and phase V, respectively. Each participant's blood pressure was measured three times at one- to two-minute intervals, and the mean was used in analysis.

### Questionnaire data

A standardized questionnaire was administered by trained interviewers to collect demographic information, lifestyle behaviors, medical history, medication use, and family history.

### Outcome and cohort follow-up

Atherosclerotic cardiovascular disease (ASCVD) encompasses coronary heart disease and ischemic stroke, according to the ICD-10 classification. In this context, coronary heart disease comprises coronary revascularization and myocardial infarction, while stroke specifically refers to ischemic stroke. The primary Outcome was first occurrence of ASCVD, the secondary Outcome was the individual outcome of coronary revascularization, myocardial infarction, and ischemic stroke; and the follow-up period started from the time of attending the third check-up, and lasted until the diagnosis of ASCVD, death, or the time of the final follow-up (December 31, 2022), which ever occurred earlier.

### Statistical methods

Statistical processing was performed using SAS 9.4 statistical software. Continuous variables with a normal distribution were expressed as mean ± standard deviation (mean ± SD) and compared between groups using analysis of variance (ANOVA). Non-normally distributed variables were presented as medians with interquartile ranges (P_25_–P_75_) and compared using the Wilcoxon signed-rank test. Categorical variables were reported as frequencies and percentages, and differences between groups were assessed using the chi-square test.

To examine the temporal and directional relationship between inflammation and metabolic disorders from multiple dimensions, cross-lagged panel analyses were conducted on cMetS and hsCRP levels during the exposure period. Standardized regression coefficients (*β*_1_ and *β*_2_) were used to evaluate directionality between the two variables, while coefficients *β*_3_ and *β*_4_ assessed the tracking correlations reflecting temporal stability. Differences between *β*_1_ and *β*_2_ were tested using Fisher's *Z* test. Pearson correlation analysis was used to assess correlations between the variables. Model fit was evaluated using the standardized root mean square residual (SRMR) and comparative fit index (CFI), with SRMR < 0.05 and CFI > 0.95 indicating a good model fit.

To evaluate the joint effect of Average cumulative hsCRP and cMetS on the risk of ASCVD, Cox proportional hazards regression models were constructed. Model 1 adjusted for age, sex, smoking status, alcohol consumption, physical activity, educational level, marital status, LDL-C, and total cholesterol. Model 2 additionally adjusted for histories of hypertension, diabetes, dyslipidemia, and family history of ASCVD. Stratified analyses were conducted to assess whether the association between exposures and ASCVD risk varied by baseline characteristics, including sex (male vs. female), age group (≤60 vs. >60 years), and 10-year ASCVD risk categories (low, intermediate, and high). On the basis of stratified analysis, the direct standardization method was applied to calculate the absolute risk of ASCVD for different groups. Sensitivity analyses were performed to test the robustness of the findings. These included: (1) excluding participants who developed an endpoint event within two years of baseline; (2) excluding those on medication; (3) excluding individuals with hsCRP >10 mg/L; (4) limiting analysis to participants who completed all three health check-ups from 2006 to 2010; and (5) competing risk models were used to account for the potential influence of non-ASCVD mortality. The Kaplan–Meier method was used to estimate cumulative incidence rates of ASCVD and its subtypes across different groups, and differences were evaluated using the log-rank test.

All statistical tests were two-sided, with a *p*-value < 0.05 considered statistically significant.

## Result

### Cross-lagged pathways and temporal relationships between inflammation and metabolic disorders

**Figure 2:**
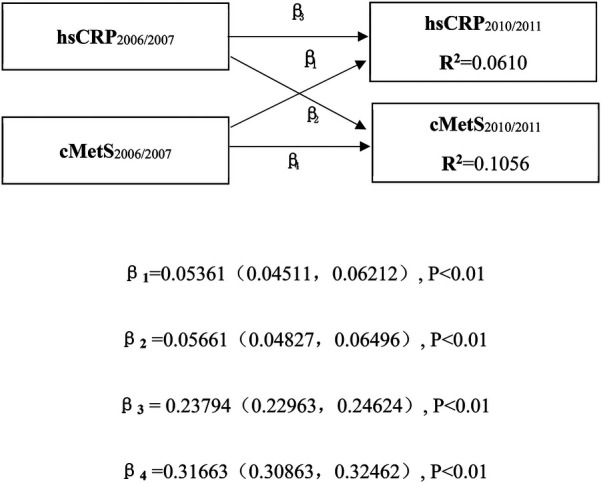
Cross-lagged path analysis of cMets and hsCRP.

A total of 62,296 participants were included in the cross-lagged analysis. Over a median follow-up of 4.05 years, and after adjustment for potential confounders, a bidirectional association was observed between hsCRP and cMetS. The path coefficient from baseline hsCRP (2006) to follow-up cMetS (2010) (β_2_ = 0.05661 95% CI: 0.04827–0.06496, *P* < 0.01) was significantly greater than the path coefficient from baseline cMetS to follow-up hsCRP (β_1_ = 0.05361, 95% CI: 0.04511–0.06212, *P* < 0.01), with a statistically significant difference between the two coefficients (*P* < 0.01). Furthermore, the tracking coefficient for *cMetS* (*β*_4_ = 0.31663, 95% CI: 0.30863–0.32462, *P* < 0.01) was greater than that for hsCRP (*β*_3_ = 0.23794, 95% CI: 0.22963–0.24624, *P* < 0.01). The variance in follow-up values explained by baseline hsCRP and cMetS (*R*^2^) was 0.0610 and 0.1056, respectively. Model fit indices (SRMR = 0.00 and CFI = 1.00) indicated acceptable model fit (Figure 2). After adjusting for confounding variables, correlation coefficients between hsCRP and cMetS at both baseline and follow-up remained significant (*P* < 0.01, Supplementary Table 1).

### Baseline characteristics of the study population

A total of 58,677 individuals were included in the final analysis, of whom 45,212 (77.05%) were male, with a mean age of (52.91 ± 11.21) years, Average CumhsCRP was 1.46 mg/L (IQR 0.76–2.88) mg/L, Average CumcMetS was 11.08 ± 1.63. Compared with participants in G1 (Low Average CumhsCRP & Low Average CumcMetS group), those in G4 (High Average CumhsCRP & High Average CumcMetS group) were more likely to be older males with higher MAP, BMI, waist circumference, fasting glucose, triglycerides, and total cholesterol, and lower HDL-C and LDL-C levels. They also had higher proportions of physical activity, married individuals, metabolic syndrome, hypertension, diabetes, dyslipidemia, and use of antihypertensive, hypoglycemic, and lipid-lowering medications. However, they had lower proportions of participants with high school education or above, smokers, drinkers, and those with a family history of ASCVD. The proportion of individuals at low and intermediate 10-year ASCVD risk decreased, while the proportion at high risk increased (all *P* < 0.01, Table 1). Average cumulative and baseline values of cMetS and hsCRP are shown in Supplementary Table 2.

**Table 1:** Comparison of baseline characteristics between different groups.

Variables	Total (*N* = 58,677)	Low Average CumhsCRP & Low Average CumcMetS (*N* = 24,714)	High Average CumhsCRP & Low Average CumcMetS (*N* = 6,069)	Low Average CumhsCRP & High Average CumcMetS (*N* = 19,826)	High Average CumhsCRP & High Average CumcMetS (*N* = 8,068)
Age, year	52.91 ± 11.21	49.87 ± 11.17	54.44 ± 11.73^a^	54.29 ± 10.29^a^	57.65 ± 10.52^a^^,^^b^^,^^c^
Male, *N* (%)	45,212 (77.05)	17,189 (69.55)	4,170 (68.71)	17,327 (87.40)^a^^,^^b^	6,526 (80.89)^a^^,^^b^^,^^c^
MAP, mmHg	99.89 ± 11.87	93.00 ± 8.47	93.71 ± 8.49^a^	107.28 ± 10.50^a^^,^^b^	107.44 ± 10.73^a^^,^^b^
BMI, kg/m^2^	25.06 ± 3.09	23.93 ± 2.77	24.60 ± 2.97^a^	26.01 ± 2.94^a^^,^^b^	26.56 ± 3.08^a^^,^^b^^,^^c^
WC, cm	88.10 ± 9.42	84.15 ± 8.63	87.22 ± 8.74^a^	91.15 ± 8.64^a^^,^^b^	93.34 ± 8.86^a^^,^^b^^,^^c^
FBG, mmol/L	5.57 ± 1.16	5.30 ± 0.83	5.35 ± 0.97^a^	5.78 ± 1.28^a^^,^^b^	6.00 ± 1.54^a^^,^^b^^,^^c^
TG, mmol/L	1.28 (0.92–1.89)	1.11 (0.83–1.53)	1.16 (0.84–1.63)^a^	1.48 (1.07–2.27)^a^^,^^b^	1.58 (1.10–2.41)^a^^,^^b^^,^^c^
TC, mmol/L	4.88 (4.30–5.49)	4.79 (4.22–5.37)	4.87 (4.28–5.50)^a^	4.93 (4.38–5.52)^a^^,^^b^	5.03 (4.44–5.72)^a^^,^^b^^,^^c^
HDL-C, mmol/L	1.47 (1.24–1.78)	1.55 (1.30–1.88)	1.51 (1.28–1.81)^a^	1.40 (1.20–1.68)^a^^,^^b^	1.36 (1.15–1.63)^a^^,^^b^^,^^c^
LDL-C, mmol/L	2.60 (2.12–3.06)	2.59 (2.18–3.00)	2.33 (1.53–2.93)^a^	2.70 (2.30–3.15)^a^^,^^b^	2.49 (1.65–3.11)^a^^,^^b^^,^^c^^,^^d^
hsCRP, mg/L	1.31 (0.70–2.97)	0.95 (0.50–1.88)	3.35 (1.40–6.20)^a^	1.25 (0.70–2.36)^a^^,^^b^	3.67 (1.70–6.49)^a^^,^^b^^,^^c^
Average CumhsCRP, mg/L	1.46 (0.76–2.88)	0.97 (0.56–1.61)	4.77 (3.68–6.79)^a^	1.23 (0.74–1.88)^a^^,^^b^	4.91 (3.77–6.91)^a^^,^^b^^,^^c^
cMetS	11.21 ± 1.81	10.00 ± 1.22	10.24 ± 1.22^a^	12.45 ± 1.44^a^^,^^b^	12.61 ± 1.48^a^^,^^b^^,^^c^
Average CumcMetS	11.08 ± 1.63	9.80 ± 0.88	9.98 ± 0.82^a^	12.41 ± 1.04^a^^,^^b^	12.58 ± 1.09^a^^,^^b^^,^^c^
2,006 MS, *N* (%)	14,297 (24.37)	1,728 (6.99)	708 (11.67)^a^	7,832 (39.50)^a^^,^^b^	4,029 (49.94)^a^^,^^b^^,^^c^
MS, *N* (%)	19,013 (32.40)	3,124 (12.64)	1,154 (19.01)^a^	9,910 (49.98)^a^^,^^b^	4,825 (59.80)^a^^,^^b^^,^^c^
Married, *N* (%)	58,388 (99.51)	24,524 (99.23)	6,039 (99.51)	19,777 (99.75)^a^	8,048 (99.75)^a^
High School or above, *N* (%)	6,086 (10.37)	3,764 (15.23)	566 (9.33)^a^	1,385 (6.99)^a^^,^^b^	371 (4.60)^a^^,^^b^^,^^c^
Current drinking, *N* (%)	19,923 (33.95)	8,267 (33.45)	1,527 (25.16)^a^	7,707 (38.87)^a^^,^^b^	2,422 (30.02)^a^^,^^b^^,^^c^
Current smoking, *N* (%)	19,550 (33.32)	7,972 (32.26)	1,788 (29.46)^a^	7,243 (36.53)^a^^,^^b^	2,547 (31.57)^b^^,^^c^
Physical exercisers, *N* (%)	40,465 (68.96)	16,690 (67.53)	4,036 (66.50)	14,199 (71.62)^a^^,^^b^	5,540 (68.67)^b^^,^^c^
Diabetes, *N* (%)	5,952 (10.14)	1,007 (4.07)	406 (6.69)^a^	2,836 (14.30)^a^^,^^b^	1,703 (21.11)^a^^,^^b^^,^^c^
Antidiabetic medications, *N* (%)	1,773 (3.02)	319 (1.29)	119 (1.96)^a^	773 (3.90)^a^^,^^b^	562 (6.97)^a^^,^^b^^,^^c^
Hyperlipidemia, *N* (%)	32,021 (54.57)	11,026 (44.61)	3,068 (50.55)^a^	12,329 (62.19)^a^^,^^b^	5,598 (69.39)^a^^,^^b^^,^^c^
Lipid-lowering medications, *N* (%)	477 (0.81)	84 (0.34)	32 (0.53)^a^	214 (1.08)^a^^,^^b^	147 (1.82)^a^^,^^b^^,^^c^
Hypertension, *N* (%)	26,644 (45.41)	4,614 (18.67)	1,323 (21.80)^a^	14,631 (73.80)^a^^,^^b^	6,076 (75.31)^a^^,^^b^^,^^c^
Antihypertensive medications, *N* (%)	5,886 (10.03)	537 (2.17)	193 (3.18)^a^	3,377 (17.03)^a^^,^^b^^,^^c^	1,779 (22.05)^a^^,^^b^^,^^c^
10-year incident risk of ASCVD					
Low risk, *N* (%)	22,558 (38.44)	14,420 (58.35)	2,527 (41.64)^a^	4,480 (22.60)^a^^,^^b^	1,131 (14.02)^a^^,^^b^^,^^c^
Medium risk, *N* (%)	15,257 (26.00)	6,362 (25.74)	1,739 (28.65)^a^	5,452 (27.50)^a^^,^^b^	1,704 (21.12)^a^^,^^b^^,^^c^
High risk, *N* (%)	20,862 (35.55)	3,932 (15.91)	1,803 (29.71)^a^	9,894 (49.90)^a^^,^^b^	5,233 (64.86)^a^^,^^b^^,^^c^
ASCVD family history, *N* (%)	7,134 (12.16)	3,173 (12.84)	663 (10.92)^a^	2,416 (12.19)^a^^,^^b^	882 (10.93)^a^^,^^b^^,^^c^

MVP, mean arterial pressure; BMI, body mass index; WC, waist circumference; FBG, fasting blood glucose; TG, triglyceride; TC, total cholesterol;HDL-C, high-density lipoprotein cholesterol; LDL-C, low-density lipoprotein cholesterol; hsCRP, high-sensitivity C-reactive protein; cMetS, continuous metabolic syndrome score; MS, metabolic syndrome; Continuous variables with a normal distribution were expressed as mean ± standard deviation (mean ± SD) and compared between groups using analysis of variance (ANOVA). Non-normally distributed variables were presented as medians with interquartile ranges (P25–P75) and compared using the Wilcoxon signed-rank test. Categorical variables were reported as frequencies and percentages, and differences between groups were assessed using the chi-square test.

a *P* < 0.05: compared with G1.

b *P* < 0.05: compared with G2.

c P < 0.05: compared with G3.

d The lower than expected LDL-C levels in G4 may be attributed to a higher prevalence of lipid-lowering medication use in this high-risk group.

### Prospective analysis of the joint impact of inflammation and metabolic disorders on ASCVD

#### Incidence density of ASCVD and cumulative ASCVD incidence in the study population

**Figure 3:**
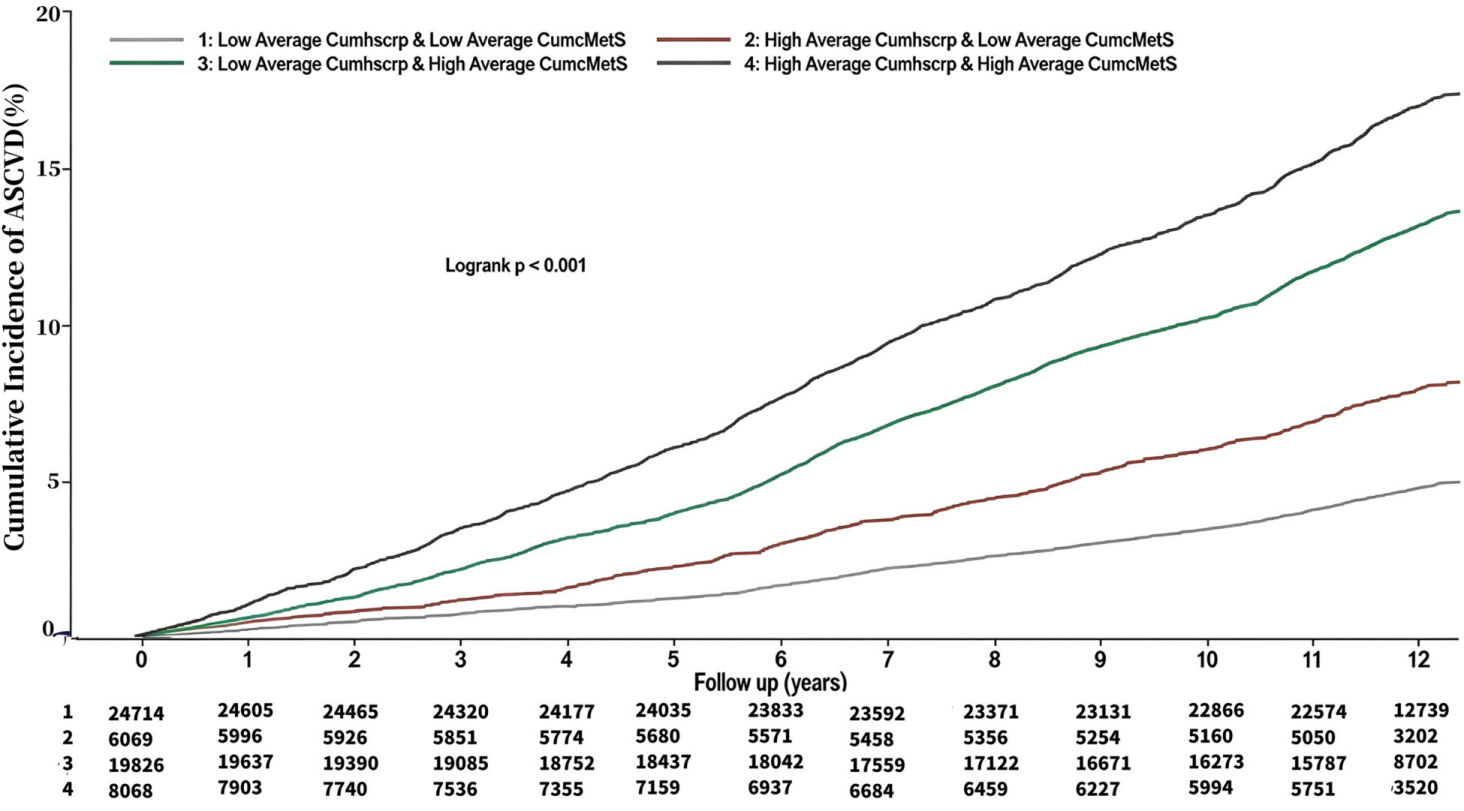
Kaplan Meier Curves for the incident of ASCVD.

**Figure 4:**
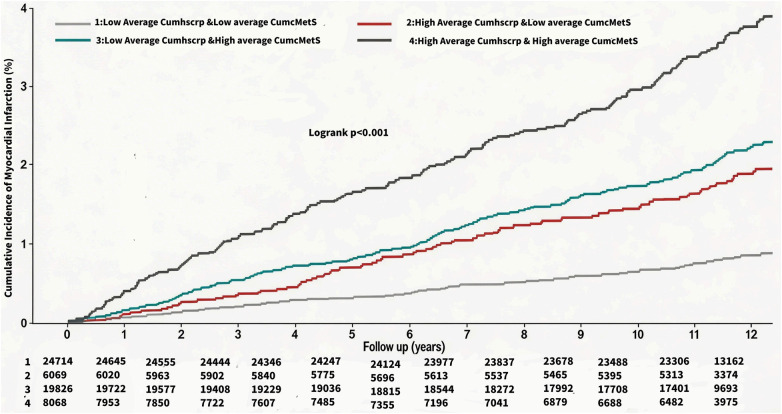
Kaplan Meier Curves for the incident of myocardial infarction.

**Figure 5:**
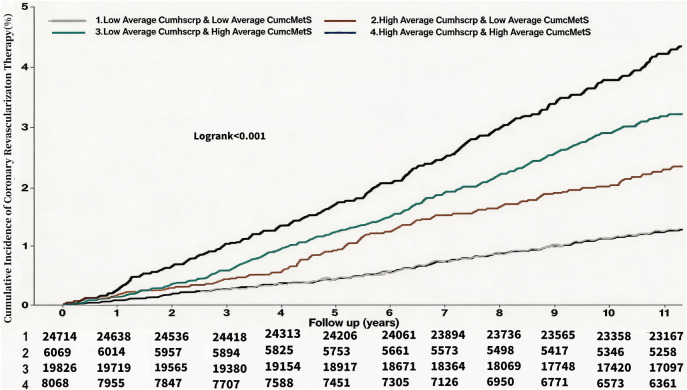
Kaplan Meier Curves for the incident of coronary revascularization therapy.

**Figure 6:**
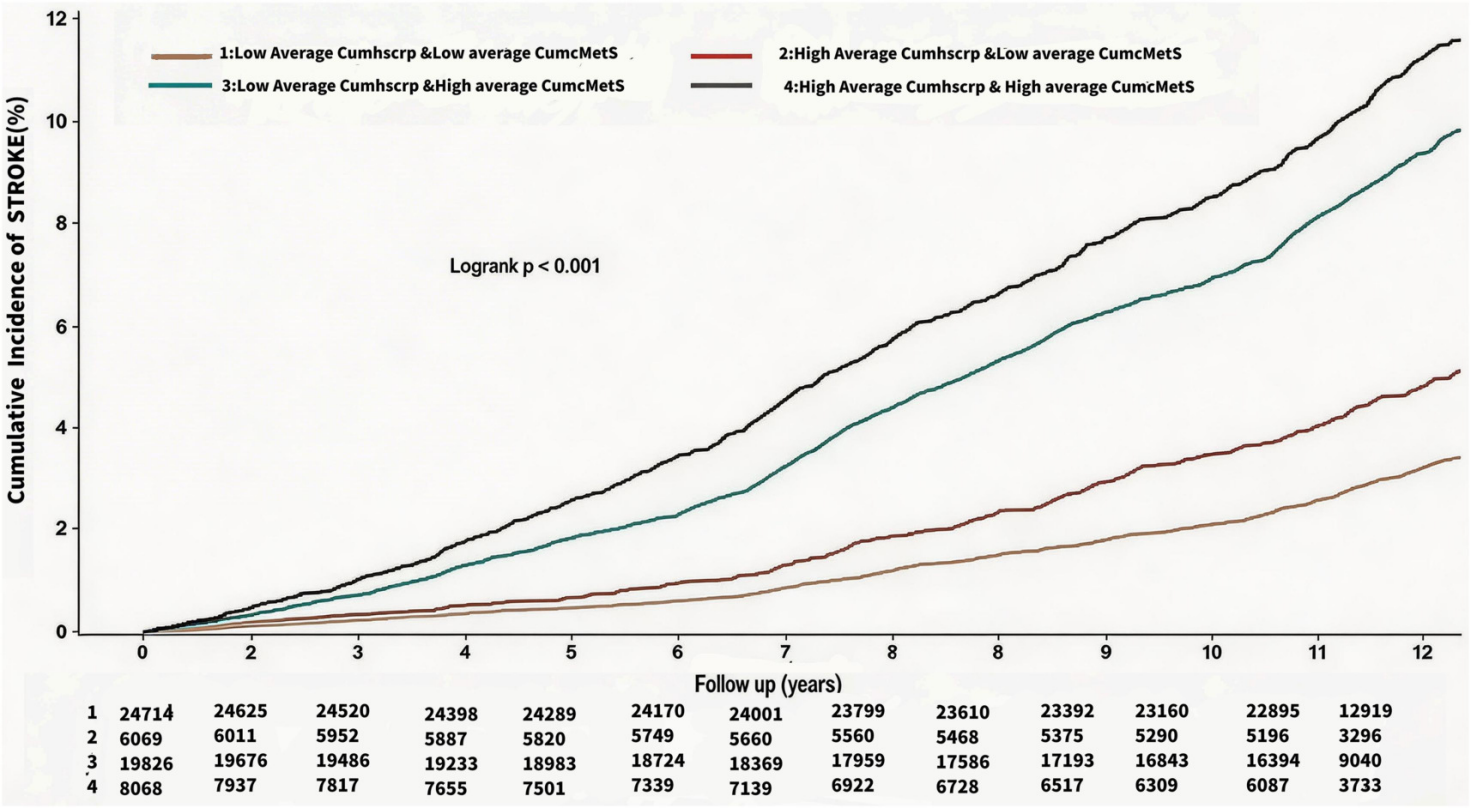
Kaplan Meier Curves for the incident of stroke.

At a mean follow-up of (11.06 ± 2.50) years, a total of 5364 patients (9.14%) developed ASCVD, and the incidence densities of ASCVD in the G1-G4 were, 4.06/thousand person-years, 6.78/thousand person-years, 11.62/thousand person-years, and 15.28/thousand person-years, respectively. Myocardial infarction occurred in 923 (1.57%) individuals, and the incidence density of myocardial infarction in the G1-G4 was 0.63/thousand person-years, 1.43/thousand person-years, 1.70/thousand person-years, and 3.00/thousand person-years. Coronary revascularization occurred in 1388 (2.37%) individuals, and the incidence densities of coronary revascularization in the G1-G4 were 1.07/thousand person-years, 2.01/thousand person-years, 2.77/thousand person-years, and 3.76/thousand person-years, respectively. Stroke occurred in 3,656 (6.23%) individuals, and the incidence densities of stroke in the G1-G4 were 2.72/thousand person-years, 4.07/thousand person-years, 8.11/thousand person-years, 9.72/thousand person-years, respectively. See Table 2. Cumulative incidence of ASCVD, myocardial infarction, revascularization, and stroke increased progressively with higher average cumulative exposures to hsCRP and cMetS, and the differences were all statistically significant as shown by the log-rank test (*P* < 0.01). See Figures 3–6.

**Table 2:** The risk of incident ASCVD upon exposure to average cumulative hsCRP and average cumulative cMetS.

Events	Combination of Average CumhsCRP and Average CumcMetS, HRs (95% CIs)				
	Low Average CumhsCRP & low Average CumcMetS	High Average CumhsCRP & low Average CumcMetS	Low Average CumhsCRP & high Average CumcMetS	High Average CumhsCRP & high Average CumcMetS	*P* for trend
ASCVD					
Event/Total	1,154/24,714	453/6,069	2,492/19,826	1,265/8,068	
IR per 1,000 person-years	4.06	6.78	11.62	15.28	
Model 1	Ref	1.47 (1.31, 1.64)	2.23 (2.08, 2.40)	2.75 (2.53, 2.99)	<0.01
Model 2	Ref	1.45 (1.30, 1.62)	1.80 (1.66, 1.95)	2.17 (1.98, 2.37)	<0.01
Myocardial infarction					
Event/Total	181/24,714	98/6,069	381/19,826	263/8,068	
IR per 1,000 person-years	0.63	1.43	1.70	3.00	
Model 1	Ref	1.99 (1.55, 2.55)	2.03 (1.70, 2.43)	3.33 (2.74, 4.05)	<0.01
Model 2	Ref	1.96 (1.53, 2.52)	1.54 (1.27, 1.88)	2.47 (2.00, 3.07)	<0.01
Coronary revascularization therapy					
Event/Total	308/24,714	137/6,069	616/19,826	327/8,068	
IR per 1,000 person-years	1.07	2.01	2.77	3.76	
Model 1	Ref	1.72 (1.40, 2.10)	2.02 (1.76, 2.32)	2.62 (2.23, 3.07)	<0.01
Model 2	Ref	1.69 (1.38, 2.07)	1.64 (1.40, 1.92)	2.06 (1.73, 2.45)	<0.01
Stroke					
Event/Total	781/24,714	276/6,069	1,771/19,826	828/8,068	
IR per 1,000 person-years	2.72	4.07	8.11	9.72	
Model 1	Ref	1.30 (1.13, 1.49)	2.33 (2.14, 2.54)	2.60 (2.35, 2.88)	<0.01
Model 2	Ref	1.28 (1.12, 1.47)	1.89 (1.72, 2.09)	2.08 (1.86, 2.32)	<0.01

Model 1: adjusted for Age, Gender, Smoking, Drinking, Physical activity, Education, Marital status, LDL, TC.

Model 2: adjusted for plus Hypertension, Diabetes, Hyperlipidemia, ASCVD family history.

#### Multivariable cox regression analysis of the impact of inflammation and metabolic disorders on ASCVD and its subtypes

The results of Cox proportional risk regression analysis of the study subjects showed that after adjusting for multiple factors such as age and gender, when compared with that in the G1, the HR (95% CI) of the risk of ASCVD in patients in the G2, G3, and G4 was 1.45 (1.30, 1.62), 1.80 (1.66, 1.95), and 2.17 (1.98, 2.37), respectively. The HR (95% CI) of the risk of myocardial infarction in patients in the G2-G4 was1.96 (1.53, 2.52), 1.54 (1.27, 1.88), and 2.47 (2.00, 3.07), respectively, compared with the G1. The HR (95% CI) risk of coronary revascularization was 1.69 (1.38, 2.07), 1.64 (1.40, 1.92), and 2.06 (1.73, 2.45) in patients in the G2-G4 compared to the G1, respectively. The HR (95% CI) of the risk of stroke in patients in the G2-G4 was1.28 (1.12, 1.47), 1.89 (1.72, 2.09), and 2.08 (1.86, 2.32), respectively, compared with the G1. Trend tests showed a significant dose–response relationship between higher cumulative hsCRP and cMetS exposure and increased ASCVD risk (*P* < 0.01, Table 2).

#### Stratified analysis of the combined effects of inflammation and metabolic disorders on ASCVD

Stratified according to sex revealed that the risk of developing ASCVD was much higher in male individuals than in female individuals (*P*_interaction_ = 0.30), and in male individuals the risk of developing ASCVD was increased by 50% (HR *=* 1.50, 95% CI: 1.33–1.70, *P* < 0.01), 84% (*HR* = 1.84, 95% CI: 1.69–2.00, *P* < 0.01), and 118% (HR = 2.18, 95% CI: 1.97–2.40, *P* < 0.01) in the G2, G3, and G4 compared with the G1, and in female individuals the risk of ASCVD was increased by 51% (HR = 1.51, 95% CI:1.21–1.88, *P* < 0.01), and 100% (HR = 2.00, 95% CI: 1.59–2.53, *P* < 0.01) in the G3, G4 compared with the G1. Stratified by age, the risk of ASCVD was found to be much higher in all groups for those aged <60 years than for those aged ≥60 years (*P*_interaction_ < 0.01), and among those aged ≥60 years the risk of ASCVD was increased by 34% (HR = 1.34, 95% CI: 1.13–1.58, *P* < 0.01), 42% (HR = 1.42, 95% CI: 1.25–1.62, *P* < 0.01), 78% (HR = 1.78, 95% CI: 1.55–2.05, *P* < 0.01) in the G2, G3, and G4 compared with the G1 and among those aged <60 years there was a 63% (HR = 1.63, 95% CI: 1.41–1.89, *P* < 0.01), 105% (HR = 2.05, 95% CI:1.86–2.27, *P* < 0.01), and 164% (HR = 2.64, 95% CI: 2.35–2.97, *P* < 0.01) increased risk of ASCVD in the G2, G3, and G4 compared with the G1.

A significant interaction was also observed between the exposure groups and 10-year ASCVD risk stratification (*P*_interaction_ < 0.01). Participants categorized as low-risk had greater relative increases in ASCVD risk than those in the intermediate- or high-risk groups. Among the low-risk population, the HRs for ASCVD in G2, G3, and G4 were1.52 (95% CI: 1.21–1.90, *P* < 0.01), 2.09 (95% CI: 1.77–2.48, *P* < 0.01), and 2.55 (95% CI: 2.02–3.23, *P* < 0.01), respectively. In the intermediate-risk group, the HRs were 1.61 (95% CI: 1.33–1.95, *P* < 0.01), 1.83 (95% CI: 1.59–2.11, *P* < 0.01), and 2.09 (95% CI: 1.74–2.50, *P* < 0.01). In the high-risk group, G3 and G4 showed significantly elevated ASCVD risks compared to G1, with HRs of 1.34 (95% CI: 1.19–1.51, *P* < 0.01) and 1.67 (95% CI: 1.48–1.90, *P* < 0.01), respectively. Full results are presented in Table 3.

**Table 3:** Stratified analysis for HR and 95% CI of ASCVD upon exposure to average cumulative hsCRP and average cumulative cMetS.

Stratified analysis		Combination of Average CumhsCRP and Average CumcMetS, HRs (95% CIs)					
			Low Average CumhsCRP & low Average CumcMetS	High Average CumhsCRP & low Average CumcMetS	Low Average CumhsCRP & High Average CumcMetS	High Average CumhsCRP & High Average CumcMetS	*P* for interaction
Gender							0.30
Male	Model 2	Event/Total	940/17,189	372/4,170	2,269/17,327	1,070/6,526	
		IR per 1,000 person-years	4.78	8.29	12.16	16.20	
		HRs (95% CIs)	Ref	1.50 (1.33, 1.70)	1.84 (1.69, 2.00)	2.18 (1.97, 2.40)	
Female	Model 2	Event/Total	214/7,525	81/1,899	223/2,499	195/1,542	
		IR per 1,000 person-years	2.44	3.69	8.01	11.62	
		HRs (95% CIs)	Ref	1.23 (0.95, 1.60)	1.51 (1.21, 1.88)	2.00 (1.59, 2.53)	
Age							<0.01
<60 years old	Model 2	Event/Total	739/20,349	247/4,214	1,598/14,338	684/4,847	
		IR per 1,000 person-years	3.10	5.12	9.98	13.05	
		HRs (95% CIs)	Ref	1.63 (1.41, 1.89)	2.05 (1.86, 2.27)	2.64 (2.35, 2.97)	
≥60 years old	Model 2	Event/Total	415/4,365	206/1,855	894/5,488	581/3,221	
		IR per 1,000 person-years	8.98	11.08	16.42	19.10	
		HRs (95% CIs)	Ref	1.34 (1.13, 1.58)	1.42 (1.25, 1.62)	1.78 (1.55, 2.05)	
10-year incident risk of ASCVD							<0.01
Low risk	Model 2	Event/Total	348/14,358	95/2,510	277/4,388	81/1,093	
		IR per 1,000 person-years	2.05	3.24	5.41	6.49	
		HRs (95% CIs)	Ref	1.52 (1.21, 1.90)	2.09 (1.77, 2.48)	2.55 (2.02, 3.23)	
Medium risk	Model 2	Event/Total	393/6,364	152/1,740	639/5,456	208/1,705	
		IR per 1,000 person-years	5.42	7.89	10.52	11.05	
		HRs (95% CIs)	Ref	1.61 (1.33, 1.95)	1.83 (1.59, 2.11)	2.09 (1.74, 2.50)	
High risk	Model 2	Event/Total	410/3,931	203/1,802	1,566/9,902	971/5,235	
		IR per 1,000 person-years	9.88	11.26	15.41	18.98	
		HRs (95% CIs)	Ref	1.18 (1.00, 1.40)	1.34 (1.19, 1.51)	1.67 (1.48, 1.90)	

Model adjusted for Age, Gender, Smoking, Drinking, Physical activity, Education, Marital status, LDL, TC, Hypertension, Diabetes, Hyperlipidemia, ASCVD family history.

Our stratified analysis was further enhanced by a detailed assessment of absolute risk. When stratified by age, the absolute risk of ASCVD was consistently higher in individuals aged ≥60 years than in those under 60 across all groups. Among participants under 60, the absolute risk was elevated to 3.63%, 5.86%, 11.15%, and 14.11% in groups G1 to G4, respectively. The corresponding absolute risks among those aged ≥60 years were 9.51%, 11.11%, 16.29%, and 18.04%.The increment in absolute ASCVD risk across groups G1 to G4 was more pronounced in the high-risk category than in the intermediate- or low-risk groups. In the low-risk group, risks rose by 2.45%, 3.79%, 7.00%, and 8.83% from G1 to G4. The intermediate-risk group had increases of 6.20%, 8.92%, 11.71%, and 12.68%, while the high-risk group had increases of 10.60%, 11.05%, 15.66%, and 18.17%. See Table 4.

**Table 4:** Absolute risk of ASCVD according to different groups.

Stratified analysis	Low Average CumhsCRP & Low Average CumcMetS	High Average CumhsCRP & Low Average CumcMetS	Low Average CumhsCRP & High Average CumcMetS	High Average CumhsCRP & High Average CumcMetS
Age				
<60 years old	3.63%	5.86%	11.15%	14.11%
≥60 years old	9.51%	11.11%	16.29%	18.04%
10-year incident risk of ASCVD				
Low risk	2.45%	3.79%	7.00%	8.83%
Medium risk	6.20%	8.92%	11.71%	12.68%
High risk	10.60%	11.05%	15.66%	18.17%

Model adjusted for Age, Gender.

#### Sensitivity analyses of the combined effects of inflammation and metabolic disorders on ASCVD

To assess the robustness of the study findings, several sensitivity analyses were conducted. Cox proportional risk regression model analyses were performed again after excluding those who had an endpoint event within 2 years from the beginning of the follow-up, those who were taking medications, and those who had hsCRP greater than 10 mg/L, respectively, and the results showed that the risk of ASCVD progressively with higher cumulative exposures to hsCRP and cMetS, similar to the results of the main analyses. The results of the death competition risk model analysis showed that compared with the G1, the risk of developing ASCVD in the G2, G3, and G4 was increased by 57% (HR = 1.57, 95% CI: 1.46–1.69, *P* < 0.01, 53% (HR = 1.53, 95% CI: 1.45–1.62, *P* < 0.01, 95% (HR = 2.17, 95% CI: 1.83–2.08, *P* < 0.01 further demonstrating the robustness of the results of this study. We also analyzed the Cox proportional risk regression model for the consecutive cohort of participants in the three health check-ups in 2006–2010, and the results obtained were consistent with the results of the main analyses. See Table 5.

**Table 5:** Sensitivity analysis for the risk of incident ASCVD upon exposure to average cumulative hsCRP and average cumulative cMetS.

Sensitivity analyses	Combination of Average CumhsCRP and Average CumcMetS, HRs (95% CIs)			
	Low Average CumhsCRP & Low Average CumcMetS	High Average CumhsCRP & Low Average CumcMetS	Low Average CumhsCRP & High Average CumcMetS	High Average CumhsCRP & High Average CumcMetS
Exclude events in the first 2 years of follow-up (*N* = 58,093)				
Event/Total	1,033/24,593	404/6,020	2,244/19,578	1,099/7,902
IR per 1,000 person-years	3.63	6.05	12.76	13.30
Model 2	Ref	1.47 (1.31, 1.65)	1.86 (1.71, 2.02)	2.19 (1.99, 2.41)
Excluding participants with hsCRP >10 mg/L (*N* = 57,212)				
Event/Total	1,154/24,700	416/5,448	2,490/19,810	1,136/7,254
IR per 1,000 person-years	4.06	6.94	11.62	15.24
Model 2	Ref	1.48 (1.32, 1.65)	1.79 (1.66, 1.94)	2.14 (1.95, 2.35)
Excluding participants with receiving medication treatment (*N* = 51,514)				
Event/Total	1,067/23,843	412/5,748	1,853/15,962	867/5,961
IR per 1,000 person-years	3.88	5.89	10.62	13.12
Model 2	Ref	1.46 (1.30, 1.64)	1.80 (1.65, 1.96)	2.19 (1.99, 2.42)
Participated in three consecutive health check ups from 2,006 to 2,010 (*N* = 43,256)				
Event/Total	823/17,741	372/4,697	1,781/14,436	991/6,382
IR per 1,000 person-years	4.00	7.15	11.33	17.63
Model 2	Ref	1.55 (1.37, 1.76)	1.79 (1.63, 1.96)	2.15 (1.94, 2.39)
The competing risk of death				
Event/Total	1,154/24,714	453/6,069	2,492/19,826	1,265/8,068
IR per 1,000 person-years	4.06	6.78	11.62	15.28
Model 2	Ref	1.57 (1.46, 1.69)	1.53 (1.45, 1.62)	1.95 (1.83, 2.08)

Model adjusted for Age, Gender, Smoking, Drinking, Physical activity, Education, Marital status, LDL, TC, Hypertension, Diabetes, Hyperlipidemia, ASCVD family history.

## Discussion

This study conducted a cross-lagged analysis in a large-scale longitudinal cohort and found a bidirectional association between inflammation (hsCRP) and metabolic disorders (cMetS), Notably, the rise in inflammation preceded the development of metabolic disorders. Furthermore, prospective cohort analyses demonstrated that joint exposure to Average cumulative hsCRP and cMetS significantly increased the risk of ASCVD, particularly among individuals younger than 60 years and those classified as having a low risk of developing ASCVD in the next 10 years.

We observed a complex reciprocal regulatory relationship between inflammation and metabolic disorders. Inflammation can initiate metabolic disorders, while metabolic disorders can in turn exacerbate inflammation through multiple biological pathways. Prior studies have indicated this mutual interaction (12). Chronic inflammation leads to the release of pro-inflammatory cytokines (e.g., TNF-α, IL-6, and IL-1β) which activate downstream signaling pathways including JNK and IKKβ/NF-κB. This disrupts the phosphorylation of insulin receptor substrates, thereby impairing insulin signaling and promoting insulin resistance (13). Conversely, in the context of obesity, Adipocyte hypertrophy triggers tissue hypoxia and cell death. Hypoxia inhibits Prolyl Hydroxylase Domain enzymes activity, preventing the ubiquitination and degradation of Hypoxia-Inducible Factor-1α and leading to its nuclear accumulation. It also impairs the mitochondrial electron transport chain, causing electron leakage and substantial generation of Mitochondrial Reactive Oxygen Species (mtROS). Moreover, severe hypoxia directly induces adipocyte necrosis, releasing Damage-Associated Molecular Patterns (DAMPs) such as ATP, Mitochondrial DNA, and uric acid crystals. Together, these DAMPs and mtROS activate the NOD-, LRR- and pyrin domain-containing protein 3 (NLRP3) inflammasome by inhibiting the AMPK pathway or promoting K^+^ efflux. This activation subsequently triggers caspase-1-mediated maturation and release of IL-1β and IL-18, which directly induce insulin resistance (14, 15). Additionally, elevated free fatty acids, particularly Saturated Fatty Acids, alter the physicochemical properties of cell membrane lipid rafts in hepatocytes and Kupffer cells, promoting Toll-like Receptor 4 (TLR4) complex assembly and activation. TLR4 recruits MyD88 (myeloid differentiation primary response 88) via its Toll/Interleukin-1 Receptor homology domain, initiating signaling that activates NF-κB. Nuclear NF-κB drives pro-inflammatory gene expression, polarizing Kupffer cells to the M1 phenotype and stimulating hepatocytes to release TNF-α and IL-6. These cytokines activate JNK and IKKβ, leading to serine phosphorylation of IRS-1/IRS-2. This impairs tyrosine phosphorylation and disrupts insulin signaling via the PI3 K/Akt pathway, ultimately inducing hepatic insulin resistance (16). These processes illustrate how inflammation and metabolism form a dynamic feedback loop that collectively regulates systemic homeostasis. Disruptions to this equilibrium contribute to the pathogenesis of several chronic diseases, including obesity, type 2 diabetes, cancer, and atherosclerosis (17). These findings demonstrate the need for clinical strategies that not only target metabolic disorders but also address the underlying inflammatory response.

Our findings also suggest that inflammation temporally precedes metabolic disorders. This temporal sequence aligns with results from earlier longitudinal studies. For instance, the Bogalusa Heart Study reported that in hyperglycemic individuals, the path coefficient from baseline hsCRP to follow-up HOMA-IR (*β*_2_ = 0.105, *p* = 0.009) was greater than that from baseline HOMA-IR to follow-up hsCRP (*β*_1_ = 0.005, *p* = 0.903) (18). Similarly, in our work, the coefficient from baseline CRP to follow-up MetS-Z was more significant (*β* = 0.032; 95% CI: 0.026–0.046, *p* < 0.001) than the reverse (*β* = 0.009; 95% CI: −0.001–0.019) (19). However, a recent meta-analysis found no causal association between CRP single nucleotide polymorphisms and the risk of metabolic syndrome (20). Lee et al. have proposed that the time lag between inflammatory and metabolic changes may occur within minutes to hours, making it difficult to capture the sequence in observational cohorts (21). Future high-resolution longitudinal studies are needed to better delineate this temporal relationship and inform clinical interventions.

This study also demonstrated that combined exposure to hsCRP and cMetS was associated with a significantly elevated risk of ASCVD. Interaction analyses revealed significant effect modification by age and 10-year ASCVD risk level (*P*_interaction_ < 0.01). Although previous studies have not specifically examined the combined impact of hsCRP and cMetS, the elevated risk associated with metabolic syndrome and high hsCRP has been well documented (22, 23). Stratified analyses further revealed that individuals under 60 years of age exhibited a higher risk of ASCVD than those aged 60 and older. This may reflect the presence of more modifiable risk factors—such as obesity, smoking, and sedentary behavior—among younger adults, and the relatively greater impact of these factors on ASCVD risk in this demographic (24). Additionally, individuals classified as low-risk based on 10-year ASCVD scores paradoxically exhibited higher ASCVD incidence under joint exposure, highlighting the need for greater clinical attention even among those deemed low risk.

This study has several important strengths. The Kailuan cohort is a large-scale prospective longitudinal study characterized by extended follow-up duration and high-quality data, thereby overcoming the inherent limitations of cross-sectional analyses. With repeated measures collected at multiple time points, we were able to employ a cross-lagged modeling approach to assess the temporal dynamics between inflammation and metabolic disorders. Moreover, we calculated the average cumulative exposure for hsCRP and cMetS, allowing for a more comprehensive representation of dynamic exposure over time and overcoming the limitations of single-time-point assessments. This approach enabled a reliable prospective evaluation of the joint impact of inflammation and metabolic disorders on ASCVD risk. Nevertheless, several limitations should be acknowledged. First, inflammation and metabolic disorders are broad constructs, and although hsCRP and cMetS serve as representative markers, they may not fully capture the underlying biological complexity. Second, although we adjusted for various potential confounders, residual confounding from unmeasured variables such as time-varying covariates, dietary patterns, sleep, and other lifestyle habits cannot be fully excluded. Third, Our analysis indicates a bidirectional relationship between inflammation and metabolic dysregulation. Although the path from inflammation to dysregulation (*β*_2_) was statistically stronger (*p* < 0.01), the actual coefficient difference was minimal (Δ = 0.003) and effect sizes were negligible. The low *R*^2^ values (<0.11) further suggest limited explanatory power of these cross-lagged effects. with the variables' own prior values being the dominant predictors.

In conclusion, this study identifies a bidirectional association between inflammation and metabolic disorders, with inflammation appearing to precede metabolic disorders. Simultaneous exposure to both Average cumulative hsCRP and cMetS substantially increases the risk of ASCVD, particularly in younger adults and those classified as having a low risk of developing ASCVD in the next 10 years. These findings emphasize the importance of integrating anti-inflammatory strategies into clinical management alongside metabolic interventions, especially in populations not traditionally classified as high risk.

## Data Availability

The raw data supporting the conclusions of this article will be made available by the authors, without undue reservation.
