# Supplementary material for: Temporal relationship between inflammation and metabolic disorders and its influence on atherosclerotic cardiovascular disease
Source: Front Cardiovasc Med. 2026 Mar 2;12:1664865. doi: 10.3389/fcvm.2025.1664865 (PMC12989371; doi:10.3389/fcvm.2025.1664865)
Supplement: Supplementary file 1 [file Datasheet1.docx]

**Supplemental Material**

**Supplemental Figure 1.** Restricted cubic splines (RCS) for the associations of cMets with the risks of ASCVD.

**Supplemental Table 1.** Pearson correlation coefficients between hsCRP and cMetS at baseline and follow-up

**Supplemental Table 2.** Comparison of Average Cumulative hsCRP and Average Cumulative cMetS to their mean value in the exposure period.


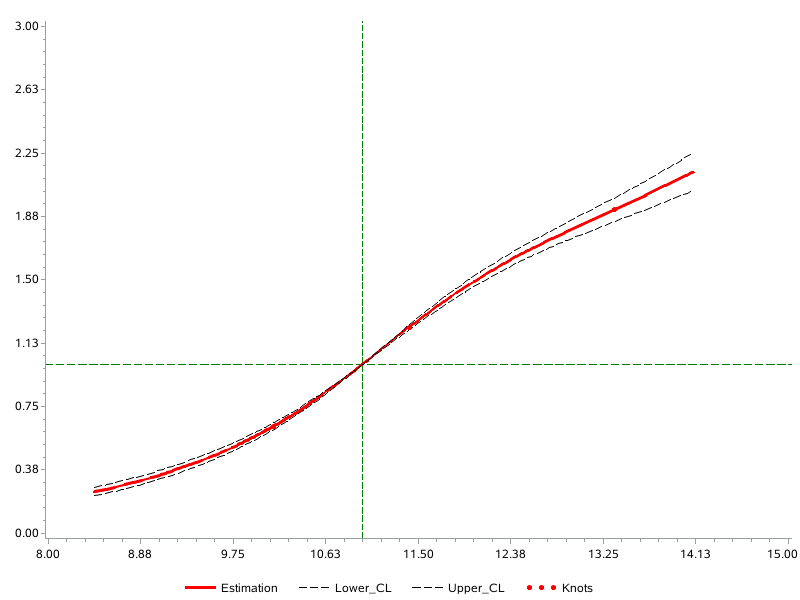


**Supplemental Figure 2.** **Restricted cubic splines (RCS) for the associations of cMets with the risks of ASCVD**

Note：P for overall association <0.01 P for non-line association<0.01

**Supplemental Table 1. Pearson correlation coefficients between scup and cMetS at baseline and follow-up**

| Variables | hsCRP2006/2007 | cMetS 2006/2007 | hsCRP2010/2011 |
| --- | --- | --- | --- |
| cMetS 2006/2007 | 0.05435 (0.046516, 0.062175) | - | - |
| hsCRP2010/2011 | 0.21706 (0.209561, 0.224527) | 0.06118 (0.053356, 0.069003) | - |
| cMetS 2010/2011 | 0.06816 (0.060338, 0.075970) | 0.28804 (0280822, 0295225) | 0.11670 (0.108946, 0.124437) |

Model 2: adjusted for Age, Gender, Smoking, Drinking, Physical activity, Education, Marital status, LDL, TC, Hypertension, Diabetes, Hyperlipidemia, ASCVD family history;

**Supplemental Table 2.** **Comparison of Average Cumulative hsCRP and Average Cumulative cMetS to their mean value in the exposure period**

|  | Mean (SD) | MAX | MIN | P25 | P50 | P75 |
| --- | --- | --- | --- | --- | --- | --- |
| cMetS at baseline | 11.21 (1.81) | 16.58 | 7.05 | 9.93 | 11.08 | 12.40 |
| Average Cumulative cMetS | 11.08 (1.63) | 15.75 | 7.46 | 9.91 | 10.98 | 12.16 |
| hsCRP at baseline | 2.29 (2.64) | 20.00 | 0.01 | 0.70 | 1.31 | 2.97 |
| Average Cumulative hsCRP | 2.40 (2.80) | 21.20 | 0.10 | 0.76 | 1.46 | 2.88 |
